# Ventricular Fibrillation Waveform Changes during Controlled Coronary Perfusion Using Extracorporeal Circulation in a Swine Model

**DOI:** 10.1371/journal.pone.0161166

**Published:** 2016-08-18

**Authors:** Raúl J. Gazmuri, Christopher L. Kaufman, Alvin Baetiong, Jeejabai Radhakrishnan

**Affiliations:** 1 Resuscitation Institute at Rosalind Franklin University or Medicine and Science, North Chicago, Illinois, United States of America; 2 Section of Critical Care Medicine at the CAPT James A. Lovell Federal Health Care Center, North Chicago, Illinois, United States of America; 3 ZOLL Medical Corporation, Chelmsford, Massachusetts, United States of America; University of Minnesota, UNITED STATES

## Abstract

**Background:**

Several characteristics of the ventricular fibrillation (VF) waveform have been found predictive of successful defibrillation and hypothesized to reflect the myocardial energy state. In an open-chest swine model of VF, we modeled “average CPR” using extracorporeal circulation (ECC) and assessed the time course of coronary blood flow, myocardial metabolism, and myocardial structure in relation to the amplitude spectral area (AMSA) of the VF waveform without artifacts related to chest compression.

**Methods:**

VF was induced and left untreated for 8 minutes in 16 swine. ECC was then started adjusting its flow to maintain a coronary perfusion pressure of 10 mmHg for 10 minutes. AMSA was calculated in the frequency domain and analyzed continuously with a 2.1 s timeframe and a Tukey window that moved ahead every 0.5 s.

**Results:**

AMSA progressively declined during untreated VF. With ECC, AMSA increased from 7.0 ± 1.9 mV·Hz (at minute 8) to 12.8 ± 3.3 mV·Hz (at minute 14) (*p* < 0.05) without subsequent increase and showing a modest correlation with coronary blood flow of borderline statistical significance (r = 0.489, *p* = 0.0547). Myocardial energy measurements showed marked reduction in phosphocreatine and moderate reduction in ATP with increases in ADP, AMP, and adenosine along with myocardial lactate, all indicative of ischemia. Yet, ischemia did not resolve during ECC despite a coronary blood flow of ~ 30% of baseline.

**Conclusion:**

AMSA increased upon return of coronary blood flow during ECC. However, the maximal level was reached after ~ 6 minutes without further change. The significance of the findings for determining the optimal timing for delivering an electrical shock during resuscitation from VF remains to be further explored.

## Introduction

There is debate on how long to perform CPR before attempting defibrillation. Some studies have suggested that a period of CPR after a long interval of untreated ventricular fibrillation (VF) helps establish myocardial conditions favorable for defibrillation [1,2]. In previous studies in a rat model of VF, Kolarova *et al*. showed that 6 minutes of CPR were needed before an electrical shock could successfully terminate VF and reestablish hemodynamically effective cardiac activity [3]. In a similar study, also in rats [4], Sun *et al*. reported changes in VF waveform by calculating the amplitude spectral area (AMSA); a method of VF waveform analysis in the frequency domain performed after fast Fourier transform in which the area under the amplitude frequency spectrum is calculated [5]. AMSA in this rat model progressively rose and reached a plateau within the same 6 minutes required for successful defibrillation [4] in the study by Kolarova *et al*. [3]. These findings were corroborated in a pig model of prolonged untreated VF of varying duration (i.e., 15, 20, 25, and 30 minutes) in which quantitative metrics of the VF waveform–including AMSA–reached a peak value after ~ 6 minutes of extracorporeal circulation (ECC) [6]. Accordingly, these studies suggest that a longer than currently recommended interval of CPR may be required to reach the myocardial conditions optimal for successful defibrillation, and that monitoring AMSA may be useful to identify myocardial “readiness” for successful defibrillation. The approach would obviate delivery of ineffective and potentially harmful electrical shocks to an ischemic myocardium [7,8]. However, other studies in swine have shown that a strategy involving 3 minutes of CPR after 8 minutes of untreated VF is less effective than immediate delivery of electrical shocks despite increases in the median VF frequency [9].

Previous pre-clinical studies have shown that changes in AMSA during chest compressions are closely related to coronary perfusion pressure [10] and therefore to the capability of the resuscitation effort to deliver oxygenated blood to the myocardium. Studies in a rat model of VF showed that increases in coronary perfusion pressure resulted in increases in VF amplitude (a component of AMSA) along with increases in myocardial creatine phosphate and decreases in myocardial lactate, indicating that VF waveform characteristics likely reflect the energy state of the myocardium [11]. Likewise, AMSA (as well as other quantitative metrics of VF waveform) was shown to correlate with myocardial ATP in swine [12].

However, no studies have examined the time course of AMSA during the resuscitation effort in relation to myocardial oxygen delivery, myocardial oxygen consumption, the resulting changes in energy metabolism, and myocardial structural changes.

In the present study, we used an open-chest swine model of VF and ECC modeling the low coronary blood flow conditions prevailing during closed-chest CPR while having direct access to the heart. The model was originally used to investigate the myocardial effects of erythropoietin given immediately before starting ECC but without analysis of AMSA [13]. The original study showed no myocardial metabolic or functional effects attributed to erythropoietin during VF and ECC but better post-resuscitation myocardial function in animals that received erythropoietin [13]. The present study reflects the unique opportunity we had to describe the time course of AMSA along with myocardial metabolic and functional changes during resuscitation without compression artifacts and under tightly controlled blood flow conditions.

## Materials and Methods

### Ethical Approval

The studies were approved by the Institutional Animal Care and Use Committee at Rosalind Franklin University of Medicine and Science and conducted according to institutional guidelines.

### Animal Preparation

Sixteen male domestic pigs (32–39 kg) obtained from Oak Hill Genetics, IL were used for the study. The animals were raised in an environmentally controlled facility and were free of Actinobacillus Pleuropneumonia, Mycoplasma Pneumonia, porcine reproductive and respiratory syndrome, and atrophic rhinitis. Upon arrival, animals were group-housed in pens in our AAALAC accredited Biological Resource Facility. Lights were set at the recommended illumination levels with a 12/12-hour light/dark cycle with the ambient temperature maintained between 61°F and 81°F. Resting mats were provided using Aspen Sani-Chip bedding from a certified vendor (Harlan Laboratories; Indianapolis, IN). General health and well-being was assessed daily by animal care technicians and veterinarians and by investigators the day of the experiment.

A detailed description of animal preparation, experimental protocol, and measurements is available in the original publication [13]. Briefly, pigs were fasted overnight, sedated with ketamine, intubated under the effect of propofol, and started on volume controlled ventilation at a tidal volume of 10 ml·kg^-1^ an FiO_2_ of 0.5 adjusting the respiratory rate to an expired end-tidal PCO_2_ between 35 and 45 mmHg. Anesthesia was provided using isoflurane (1.75% to 2.75%) and a 1:1 mixture of nitrous oxide and oxygen titrated to attain a surgical plane. Animals were instrumented through peripheral vascular access with an aortic catheter, a thermodilution balloon-tipped pulmonary artery catheter, and an angiographic catheter advanced into the great cardiac vein. For ECC, cannulas were advanced from the left external jugular vein into the right atrium for blood withdrawal and into the left carotid artery for blood return. Heparin (100 units·kg^-1^) was then injected into the right atrium and cannulas connected to a membrane oxygenator system primed with ~750 ml of 6% hetastarch. During ECC, the oxygenator was supplied with a 95% O_2_ and 5% CO_2_ gas mixture at a constant rate of 3 l·min^-1^. The heart was then accessed through a midline sternotomy and instrumented to assess left ventricular pressures and the left anterior descending (LAD) coronary blood flow.

A lead II electrocardiogram (ECG) was recorded through defibrillation pads attached to the each side of the chest wall using an Agilent defibrillator (HeartStream XL) in the initial 8 experiments and through a standard limb ECG configuration using a ZOLL defibrillator (E series) in the last 8 experiments.

### Experimental Protocol

Anesthesia was discontinued and VF induced by delivering an alternating current to the epicardium (1–10 mA for ~ 1–3 s) without observing spontaneous reversal to an organized rhythm in any instance. Ventilation was discontinued and VF maintained untreated for 8 minutes. ECC was then started and its flow adjusted to generate a coronary perfusion pressure (CPP) of 10 mmHg (simulating average quality CPR) for 10 minutes (low-flow ECC). After 18 minutes of VF including 10 minutes of ECC, a single, low energy (5-J), biphasic electrical shock was delivered to the epicardium to assess whether erythropoietin could lower the defibrillation threshold [13]. Immediately after the shock was delivered, positive pressure ventilation and anesthesia were resumed and the ECC flow increased adjusted to maintain a mean aortic pressure at 40 mmHg (high-flow ECC) and ensuring the duration of low-flow ECC was comparable in all animals. Additional electrical shocks were given if VF persisted at 60-second intervals escalating their energy to 10, 20, and 30 J. ECC was continued after return of cardiac activity as needed to maintain the mean aortic pressure at 40 mmHg for the 120 minutes post-resuscitation observation interval. Pigs were randomized to receive erythropoietin (1,200 U·kg^-1^ bolus, Procrit^®^, Janssen Biotech, Horsham, PA) or 0.9% NaCl into the right atrium before starting ECC. The findings on the effects of erythropoietin have been reported [13] and occurred post-resuscitation not during resuscitation from VF; hence, we combined the erythropoietin and vehicle-control treated pigs for the present study. The pig was euthanized at the end of 120 minutes while receiving general anesthesia by intravenous injection of euthanasia solution.

### Measurements

Blood was collected from the aorta and great cardiac vein measuring O_2_ content (ml/dl) and lactate concentration. LAD blood flow was reported as percentage of baseline and used to calculate myocardial O_2_ delivery (MDO_2_) and consumption (MVO_2_) and myocardial lactate consumption and production. The coronary perfusion pressure was calculated during ECC as the difference between the mean aortic and the mean right atrial pressures. The heart was imaged by epicardial echocardiography obtaining a four-chamber view from the apex and a transverse view from the anterior left ventricular wall and used to calculate left ventricular wall thickness and left ventricular volumes.

The myocardium was sampled at baseline and during VF while on ECC (VF-ECC) using a 5.0-mm punch biopsy tool yielding 30–40 mg of cylindrical specimens that encompassed approximately 80% of the wall thickness. The samples were obtained from the anterior left ventricular wall starting at the apex and moving toward the base in a zigzag pattern to avoid vascular injury that could compromise blood supply to the region of the subsequent sample. Samples were immersed in liquid N_2_ within 10 seconds, stored at -80°C, and subsequently processed for creatine, phosphocreatine, adenosine, adenosine monophosphate, adenosine diphosphate (ADP), and adenosine triphosphate (ATP) using reverse-phase high-performance liquid chromatography (System Gold, Beckman, and 32 Karat Software 5.0, Fullerton, CA) along with lactate concentration as previously described [14].

The VF signal was analyzed in the frequency domain after fast Fourier transform and AMSA calculated as the summed product of frequency and square root of power (amplitude, A) at that frequency (F) from 2 to 48 Hz [AMSA = Σ(Ai * Fi)]. This calculation was performed every 0.5 seconds with a 2.1-second Tukey window. The individual AMSA values were averaged over 60 seconds for the purpose of this analysis. Pulsatile artifacts that precluded AMSA analysis occurred during high-flow ECC in the first 8 experiments when the ECG was obtained through defibrillation pads attached to the chest wall. No such artifacts occurred during low-flow ECC and when the ECG was obtained through limb electrodes. Accordingly, the analysis herein reported included 16 experiments during low-flow ECC and 8 experiments during high-flow ECC. In all these instances, the raw ECG signal was processed similarly and therefore the VF waveform analysis values for all experiments could be pooled.

### Statistical Analysis

Descriptive statistics are presented as mean ± SEM in figures and mean ± SD elsewhere. SigmaPlot 11.0 (Systat Software, Point Richmond, CA) was used for all the statistical analyses. Changes over time were assessed by repeated measures ANOVA. Simple linear regression was used to assess the variability of AMSA over time. A two-tail value of p<0.05 was considered significant.

## Results

**Fig 1:**
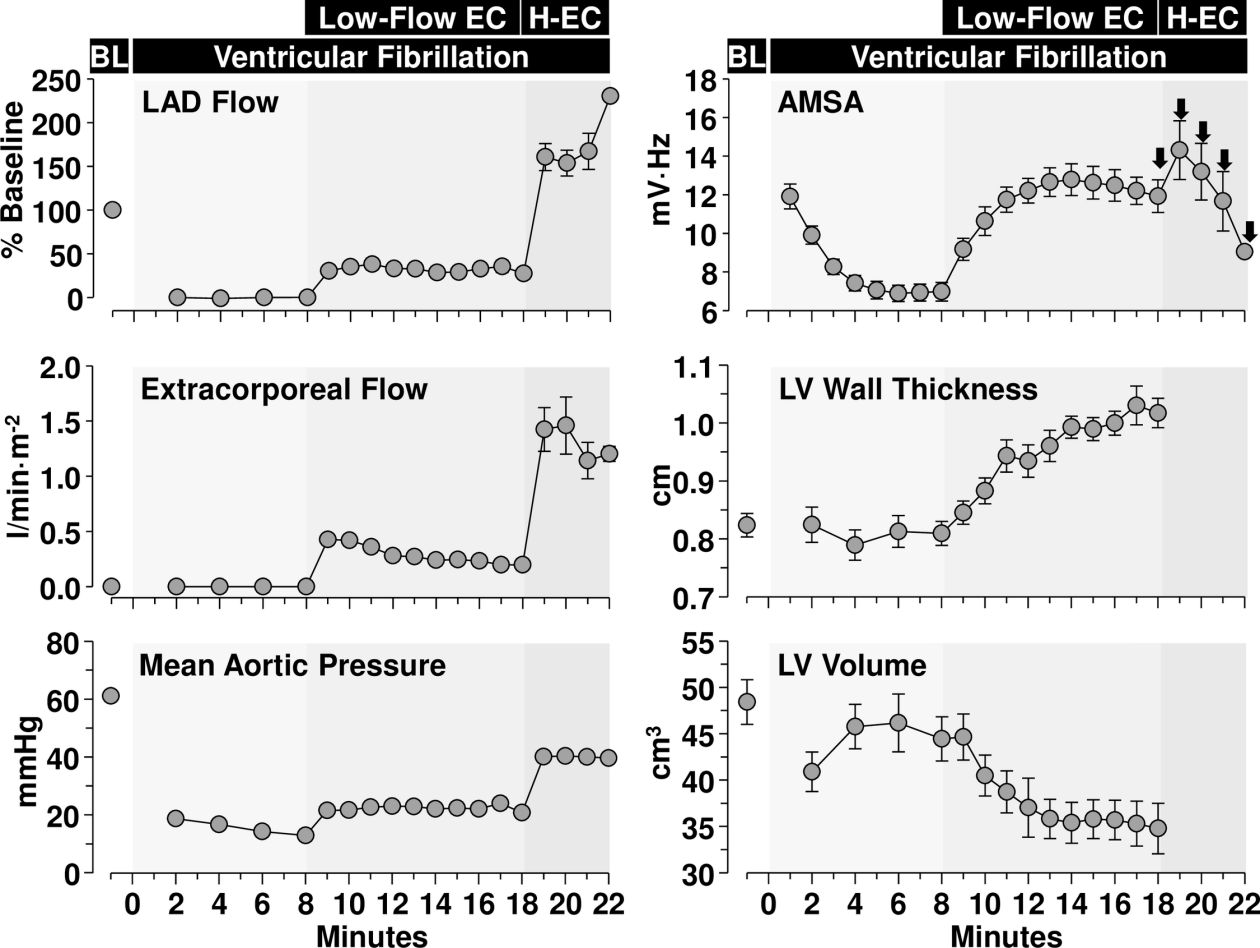
Effects of ventricular fibrillation and simulated resuscitation using extracorporeal circulation (EC) on the ventricular fibrillation amplitude-spectral area (AMSA) and left ventricular (LV) volume and wall thickness. The EC flow was first titrated to maintain a coronary perfusion pressure of 10 mmHg (not shown) from minute 9 to 18 (Low-Flow EC) and then increased during the defibrillation effort and the post-resuscitation interval to generate a higher (H) mean aortic pressure of 40 mmHg (H-EC). Arrows signal the time of the electrical shocks.

AMSA significantly declined during the initial 8 minutes of untreated VF from 11.9 ± 2.6 mV·Hz (at minute 1) to 6.9 ± 1.7 mV·Hz (at minute 6) (*p* < 0.05) and remained at this level until ECC was started. Low-flow ECC–titrated to generate a CPP of 10 mmHg and resulting in an LAD flow of ~ 30% of baseline–significantly increased AMSA from 7.0 ± 1.9 mV·Hz (at minute 8) to 12.8 ± 3.3 mV·Hz (at minute 14) (*p* < 0.05). Thereafter, AMSA plateaued (or slightly declined) (Fig 1). AMSA and LAD flow averaged during low-flow ECC correlated with borderline statistical significance (r = 0.489, *p* = 0.0547). The 5-J electrical shock delivered at minute 18 failed in each instance to terminate VF. High-flow ECC resulted in an increase in LAD to ~ 180% of baseline and was associated with an increase in AMSA from 11.9 ± 3.2 mV·Hz (at minute 18) to 14.3 ± 4.3 mV·Hz (at minute 19). However, from minute 19 to minute 22 while defibrillation was being attempted and high-flow ECC maintained, AMSA declined precipitously to 9.1 ± 0.1 mV·Hz at minute 22 (Fig 1). Yet, the decline of AMSA did not preclude successful defibrillation in each instance within 4 minutes of high-flow ECC.

During the interval of VF and ECC, the left ventricular wall progressively thickened and the left ventricular volume progressively decreased (Fig 1) without changes in left ventricular pressure (data not shown) demonstrating reductions in left ventricular distensibility. Left ventricular wall thickness and left ventricular volumes measured in diastole at minute 5 of the post-resuscitation phase–after all hearts had been successful defibrillated–were not different than measurements obtained during VF in the last minute of low-flow ECC demonstrating post-resuscitation diastolic dysfunction.

**Fig 2:**
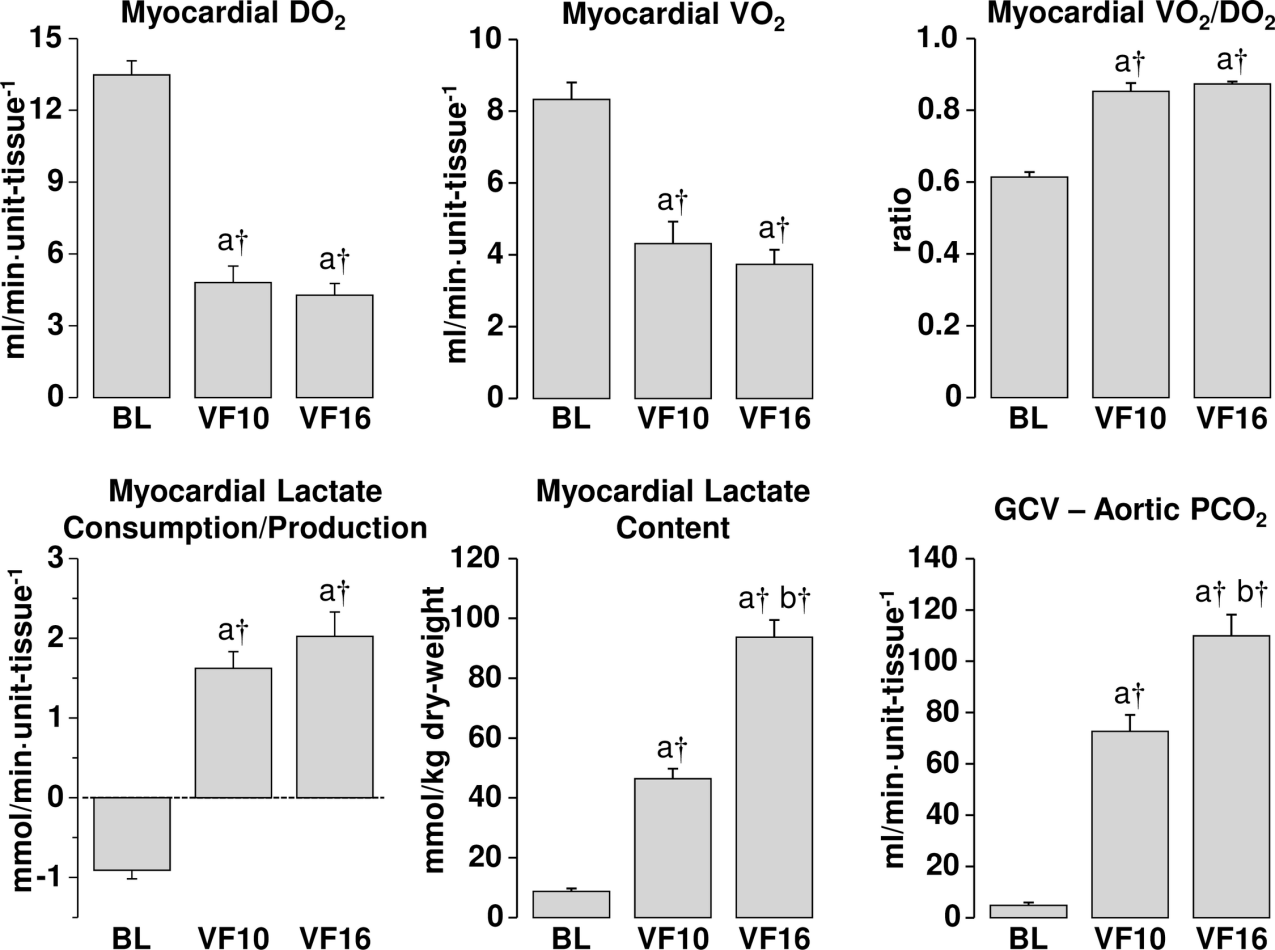
Myocardial measurements at baseline (BL) and during ventricular fibrillation (VF), at 10 and 16 minutes while on low-flow extracorporeal circulation simulating the hemodynamic conditions of closed-chest CPR. DO_2_, oxygen delivery; VO_2_, oxygen consumption; GCV, great cardiac vein. Mean ± SEM of 16 experiments. Data was analyzed by one-way repeated measures ANOVA and differences shown after Holm-Sidak method for multiple pairwise comparisons. a *vs* BL, b *vs* VF10; *p ≤ 0.05, †*p* ≤ 0.001.

**Fig 3:**
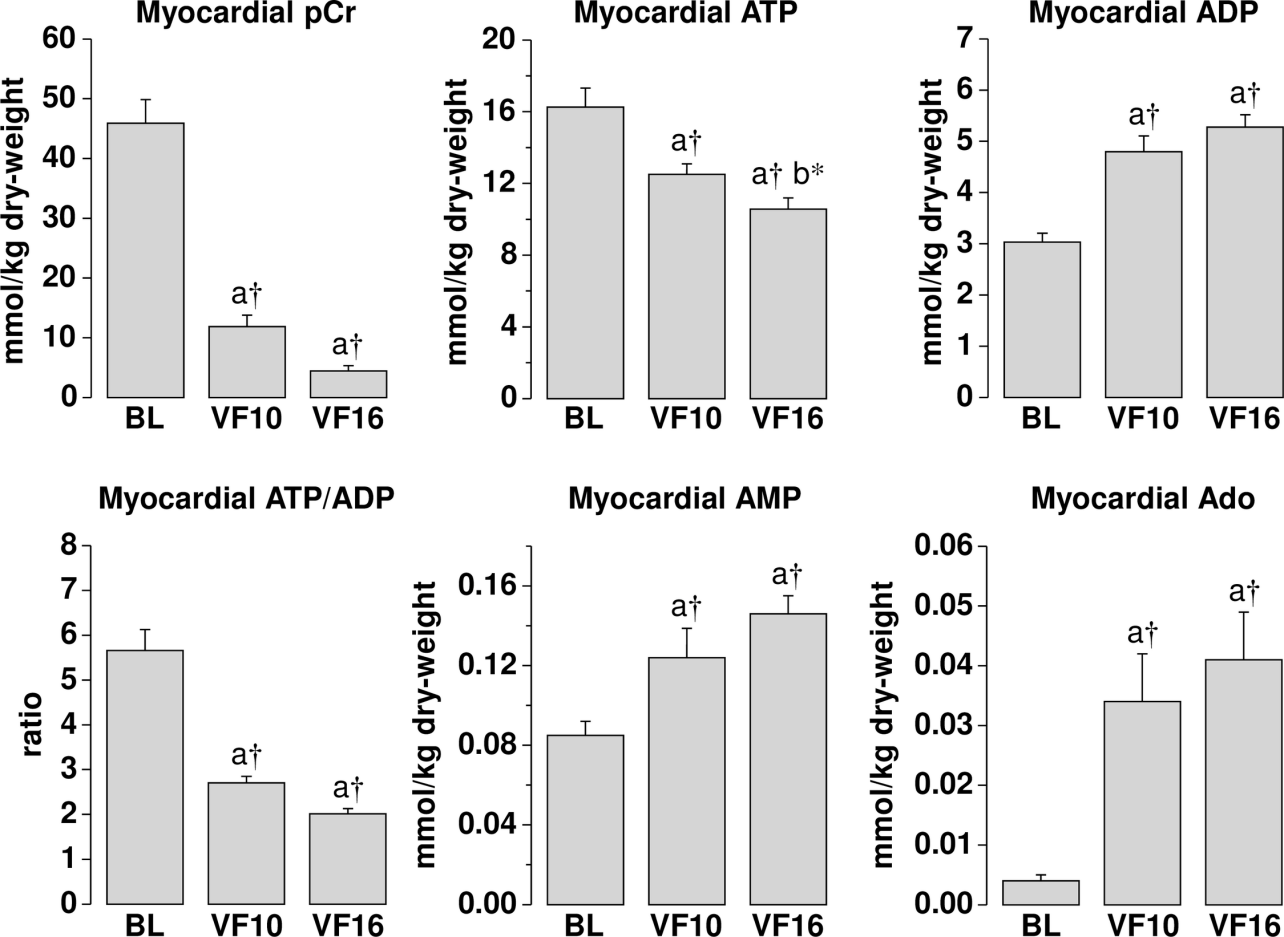
Myocardial measurements at baseline (BL) and during ventricular fibrillation (VF), at 10 and 16 minutes while on low-flow extracorporeal circulation simulating the hemodynamic conditions of closed-chest CPR. pCr, phosphocreatine; ATP, adenosine triphosphate; ADP, adenosine diphosphate; AMP, adenosine monophosphate; Ado, adenosine. Data was analyzed by one-way repeated measures ANOVA and differences shown after Holm-Sidak method for multiple pairwise comparisons. a *vs* BL, b *vs* VF10; *p ≤ 0.05, †*p* ≤ 0.001.

Low-flow ECC resulted in a myocardial O_2_ delivery of 36% (at VF 10 minutes) and 32% (at VF 16 minutes) of baseline; a level that prompted maximal myocardial O_2_ extraction at 85% and 87%, respectively, but failed to maintain myocardial O_2_ consumption at levels required to meet metabolic demands, evidenced by a shift from lactate consumption at baseline to lactate production at VF 10 minutes and VF 16 minutes. These abnormalities were accompanied by progressive myocardial accumulation of lactate and progressive increase in the coronary PCO_2_ gradient attaining levels at VF 16 minutes that were significantly higher than those observed at VF 10 minutes (Fig 2). Concurrently, there was marked reduction in myocardial phosphocreatine accompanied by mild reductions in ATP. However, the decreases in ATP levels–like the changes in myocardial lactate and coronary PCO_2_ gradient–were progressive with levels at VF 16 minutes significantly lower than those at VF 10 minutes (Fig 3). As expected, levels of breakdown products including ADP, AMP, and adenosine were all increased (Fig 3).

**Fig 4:**
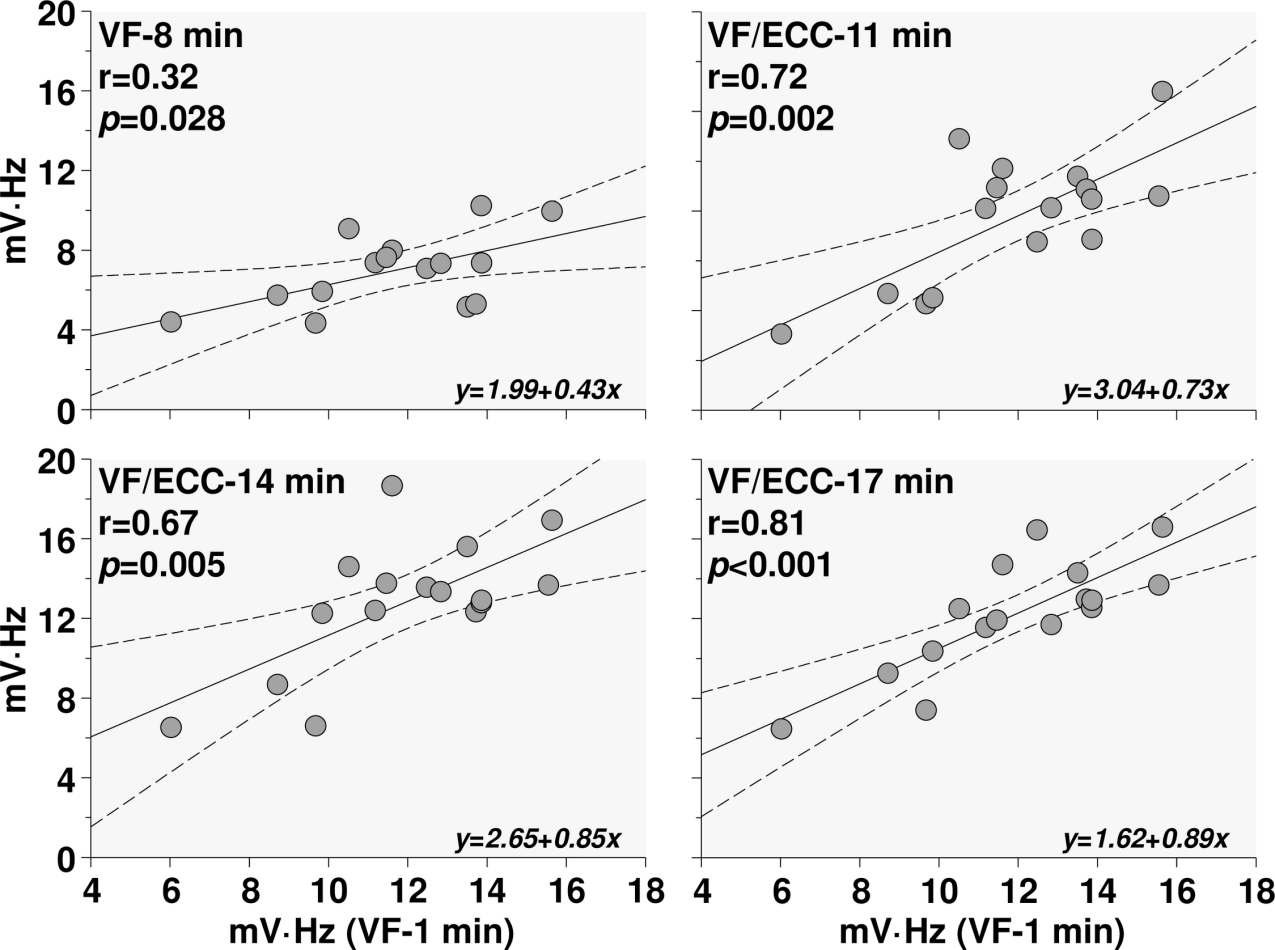
Correlations between AMSA measured 1 minute after induction of VF (VF-1 min) and measurements after 8 minutes of untreated VF (VF-8 min) and at 11, 14, and 17 minutes while ECC was on.

AMSA presented with wide variability not attributable to cardiac arrest or resuscitation. As shown in Fig 4, the variability observed shortly after induction of VF (at minute 1, ranging from 6.0 to 15.6 mV·Hz) was highly correlated with the variability observed during untreated VF at minute 8 and during low-flow ECC at minutes 11, 14, and 17.

## Discussion

We described the time course of AMSA in relation to myocardial metabolic changes under tightly controlled hemodynamic conditions using ECC to deliver a systemic blood flow representative of average CPR. AMSA progressively rose during ECC and reached a maximum after approximately 6 minutes. Thereafter, AMSA remained largely unchanged under conditions in which the severity of myocardial ischemia persisted and changes consistent with reperfusion injury ensued. The shock delivered at the end of low-flow ECC was uniformly unsuccessful. Upon switching to high-flow ECC, AMSA exhibited a transient increase followed by rapid decline to levels below those observed at the end of low-flow ECC but without precluding successfully defibrillation. The study therefore provides new insights on factors that determine AMSA during CPR and identifies additional areas for further research.

### AMSA Time Course

During the interval of no-flow VF, AMSA gradually declined reaching a nadir after 4 to 6 minutes reflecting the metabolic effects of global myocardial ischemia curtailing the energy-generating processes required for action potential generation. This time window exceeded by a only a few minutes the time window during which an electrical shock can successfully terminate VF and reestablish cardiac activity without intervening CPR; which has been termed the electrical phase of cardiac resuscitation [15]. Thereafter, CPR is required to reestablish the myocardial metabolic conditions for successful resuscitation.

During the interval of low-flow ECC, AMSA rose gradually coincident with the reintroduction of oxygenated blood and the restart of energy-generating processes. The time course of AMSA was similar to that observed in rat experiments [3,4] reaching a plateau after approximately 6 minutes of low-flow ECC with an AMSA value similar to the AMSA value observed after the first minute of untreated VF (Fig 1). In one of the rat studies, shocks were delivered when the AMSA value reached 10 mV-Hz resulting in fewer defibrillation attempts and a CPR duration of approximately 6 minutes to achieve return of spontaneous circulation (ROSC), shorter than a guideline-based treatment protocol [3,4]. In an earlier rat study [3], 6 minutes of CPR were also required for electrical shocks to attain ROSC with earlier shock being uniformly unsuccessful. In both of these rat studies, the CPP was maintained above resuscitability thresholds.

Accordingly, our current study along with the previous rat studies suggest that successful defibrillation requires that CPR be hemodynamically effective and that such effectiveness by maintained over time until optimal myocardial conditions are reached–which could be identified by AMSA–before delivering an electrical shock. In studies similar to ours, also in swine using ECC, Salcido *et al*. [6] suggested that the time to maximal VF-derived indices–including AMSA–was influenced by prior duration of untreated VF, with AMSA reaching its maximum after 8 to 9 minutes of ECC when the preceding interval of untreated VF was 30 minutes.

### Underlying Myocardial Changes

The coronary blood flow generated during low-flow ECC corresponded to ~ 30% of the baseline flow. Such blood flow, which reflects the expected blood flow during closed-chest CPR, failed to meet the metabolic demands of the fibrillating myocardium despite maximal oxygen extraction evidenced by the reduced oxygen consumption, persistent lactate production, and elevated veno-arterial coronary PCO_2_ gradient, all indicative of anaerobic metabolism not abated by low-flow ECC. The energy nucleotides pattern was also indicative of inadequate oxygen delivered; evidenced by low phosphocreatine demonstrating the inability of mitochondria to meet the energy requirements. These myocardial abnormalities were observed at minute 2 of low-flow ECC but persisted or even worsened during the ensuing 8 minutes of low-flow ECC indicating that the level of coronary blood flow–that we had considered representative of average CPR–was not sufficient to reverse myocardial ischemia during VF in this swine model.

It is conceivable that a narrow time window exists for successful defibrillation once the “optimal” myocardial conditions are reached for a given myocardial blood flow level during CPR, and that delays in shock delivery may reduce its effectiveness. It is important to emphasize that reperfusion not only delivers oxygen, but also triggers reperfusion injury with generation of reactive oxygen species and sodium-driven cytosolic and mitochondrial calcium overload. An important manifestation of reperfusion injury during resuscitation from VF is progressive myocardial wall thickening with reductions in left ventricular volumes but without changes in left ventricular pressures as observed in the current experiments consistent with decreases in left ventricular distensibility [14,16]. We have previously linked these myocardial abnormalities to cytosolic and mitochondrial Ca^2+^ accumulation [17] compromising mitochondrial bioenergetic function [14]. The changes in left ventricular volumes seemed to have paralleled the time course of AMSA. Because myocardial size and mass can impact the amplitude of the ECG, these findings raised the possibility that VF waveform metrics could have been influenced by changes in left ventricular volumes. In a similar swine model, we reported that inhibition of the sarcolemmal sodium-hydrogen exchanger isoform-1 (NHE-1) attenuate these myocardial abnormalities coincident with preservation of mitochondria bioenergetic function [14].

### Shock Success during Low-Flow and High-Flow ECC

We designed the study to deliver the first electrical shock after 10 minutes of low-flow ECC at a low energy of 5-J to assess whether erythropoietin could have lowered the defibrillation threshold, which in similar previous studies resulted in 20% shock success. This was not the case and the 5-J shock uniformly failed to terminate VF. Yet, we cannot exclude that VF could have been successfully terminated during low-flow ECC had we escalated the electrical shock energy or had we delivered the low-energy electrical shock after 6 minutes of low-flow ECC when AMSA ceased to increase. Delivery of electrical shocks of higher energy during high-flow ECC eventually terminated VF and reestablished cardiac activity in each instance. High-flow ECC maintained the mean aortic pressure at 40 mmHg and resulted in a coronary blood flow of ~ 180% of baseline, likely the result of reactive hyperemia [18,19].

Intriguingly, AMSA first increased with the higher coronary blood flow but rapidly declined to levels below those observed with low-flow ECC, yet, the declining AMSA did not preclude successful defibrillation. Repetitive electrical shocks have been associated with additional myocardial injury induced by Ca^2+^ overload and electroporation [8,20,21], raising the possibility that the AMSA threshold for predicting successful defibrillation might change after defibrillation attempts.

### Intrinsic AMSA Variability

We observed broad AMSA variability at each time point, despite the highly controlled hemodynamic conditions, which could not be explained by variability in the metabolic parameters investigated (data not shown). The variability of AMSA at minute 1 was highly correlated with the variability at subsequent time points (Fig 4) consistent with intrinsic factors determining an AMSA value unique to each individual and which should be considered when attempting to identify values predictive of shock success. The very tight hemodynamic conditions precluded assessing the dependency of AMSA on the various hemodynamic and metabolic parameters. Although we observed a modest positive correlation between AMSA and coronary blood flow, most of the individual parameters (e.g., myocardial lactate, phosphocreatine, ATP, ATP/ADP ratio) were not correlated.

## Conclusions

The present study served to characterize the time course of AMSA showing its broad dependency on coronary blood flow. It was interesting to note that an AMSA plateau was reached after approximately 6 minutes, similar to the time required in rats to reach conditions optimal for successful defibrillation. Because of the very tight hemodynamic conditions, the study did not allow to examine specific myocardial determinants of AMSA. It is intriguing that AMSA declined after increasing ECC flow coincident with the hyperemic reaction that follows return of normal CPP and with the delivery of electrical shocks. Further work in a similar animal model in which the levels of CPP are varied would shed light on the various myocardial determinants of AMSA.

## Supporting Information

S1 File Underlying Data. (XLSX)
